# Understanding N timing in corn yield and fertilizer N recovery: An insight from an isotopic labeled-N determination

**DOI:** 10.1371/journal.pone.0192776

**Published:** 2018-02-20

**Authors:** Silas Maciel de Oliveira, Rodrigo Estevam Munhoz de Almeida, Ignacio A. Ciampitti, Clovis Pierozan Junior, Bruno Cocco Lago, Paulo Cesar Ocheuze Trivelin, José Laércio Favarin

**Affiliations:** 1 Department of Crop Science, University of São Paulo, Luiz de Queiroz College of Agriculture, Piracicaba, São Paulo, Brazil; 2 Brazilian Corporation of Agricultural Research, Fishery and Aquiculture, Palmas, Tocantins, Brazil; 3 Department of Agronomy, Kansas State University, Manhattan, Kansas, United States of America; 4 Paraná Federal Institute of Education, Science and Technology, Palmas, Paraná, Brazil; 5 University of São Paulo, Center of Nuclear Energy in Agriculture, Piracicaba, São Paulo, Brazil; University of Oklahoma, UNITED STATES

## Abstract

Early fertilizer nitrogen (N) application on cover crops or their residues during the off-season is a practice adopted in Brazil subtropical conditions under no-tillage corn (*Zea mays* L.) systems. However, the effect of early N application on yield, plant N content, and N recovery efficiency (NRE) for corn is not yet well documented. Five fertilizer N timings in an oat-corn system were evaluated in two studies utilizing an isotopic-labeled N determination, ^15^N isotope. The N fertilization timings were: (i) oat tillering, (ii) 15 days before corn planting time, over the oat residues, (iii) at corn planting time, (iv) in-season at the three-leaf growth stage (V3), and (v) in-season split application at V3 and six-leaf (V6) growth stages. Based on the statistical analysis, the N fertilization timings were separated into three groups: 1) N-OATS, designated to N applied at oat; 2) N-PLANT, referred to pre-plant and planting N applications; and 3) N-CORN, designated to in-season corn N applications. Corn yield was not affected by the N fertilization timing. However, the N-CORN N fertilization timings enhanced NRE by 17% and 35% and final N recovery system (plant plus soil) by 16% and 24% all relative to N-OATS and N-PLANT groups, respectively. Overall, N-OATS resulted in the largest N derived from fertilizer (NDFF) amount in the deeper soil layer, in overall a delta of 10 kg N ha^-1^ relative to the rest of the groups. Notwithstanding corn yield was not affected, early N fertilization under subtropical conditions is not a viable option since NRE was diminished and the non-recovery N increased relative to the in-season N applications.

## Introduction

Improving nitrogen (N) recovery efficiency (NRE) could assist reducing N inputs (e.g. fertilizer) and N environmental footprint in agricultural systems. Among management practices affecting NRE, adoption of conservation tillage [1, 2] and timing of nutrient application, less asynchrony between soil N supply and plant N demand [3,4], should be highlighted. For cereals, NRE was up to 30% greater under no-tillage relative to conventional tillage [5, 6]. While most recent adoption of conservation tillage practices improved N management, timing of N application is often affected by labor constraints or logistical concerns [7, 8, 9]. For corn (*Zea mays* L.), previous ^15^N studies documented NRE ranging from 30% to 55% regarding the timing of the fertilizer N application [2, 10–13].

In Brazil, subtropical conditions allow for cover crop cultivation in the fall (off-season). For those farming systems, the N fertilization practice has been applied to cover crop or its residues, after desiccation with herbicides was implemented [14, 15]. In this N fertilization strategy, the crop will be receiving N directly from the fertilizer and also from the N immobilized but later released by the residues of the cover crop. In temperate climate, early N application in cereals was reflected by reductions on yield and fertilizer use efficiency relative to in-season N applications [16–18]. Nonetheless, in subtropical conditions there is scarce information on this topic, primarily on comparing fertilization N timing and its effects on crop yields as when N is applied to the precedent crop, at corn planting, and in-season.

Few studies evaluated early N application under subtropical conditions but reported comparable yields under different N fertilization timings [19–21]. Early N could be equally effective as in-season N applications since N could promote decomposition of residues and release of N [22, 23]. On the other hand, N fertilizer can remain for a longer period of time in the field and be subjected to loss [24, 25] and microbial immobilization [26]. Therefore, soil N supply might be favored and plant N demand could be fulfilled in a larger proportion by the indigenous soil N supply coming from the mineralization process [27, 28].

The overall objective of this study is to provide a better understanding of the effect of fertilizer N timing and their recovery efficiency for corn preceded by a cover crop. Following this rationale, three main goals were pursued across all fertilizer N application timings: 1) study the effect of fertilizer N timing application on corn grain yield, plant N content, and NRE; 2) evaluate the relationship of N derived from fertilizer (NDFF) with biomass and plant N content, and determine the fertilizer N fraction at varying fertilization N timing; and 3) describe the proportion of fertilizer ^15^N recovery accounted for all plant fractions and soil N pool relative to their contributions to plant N content for corn crop.

## Materials and methods

### Site description

**Fig 1:**
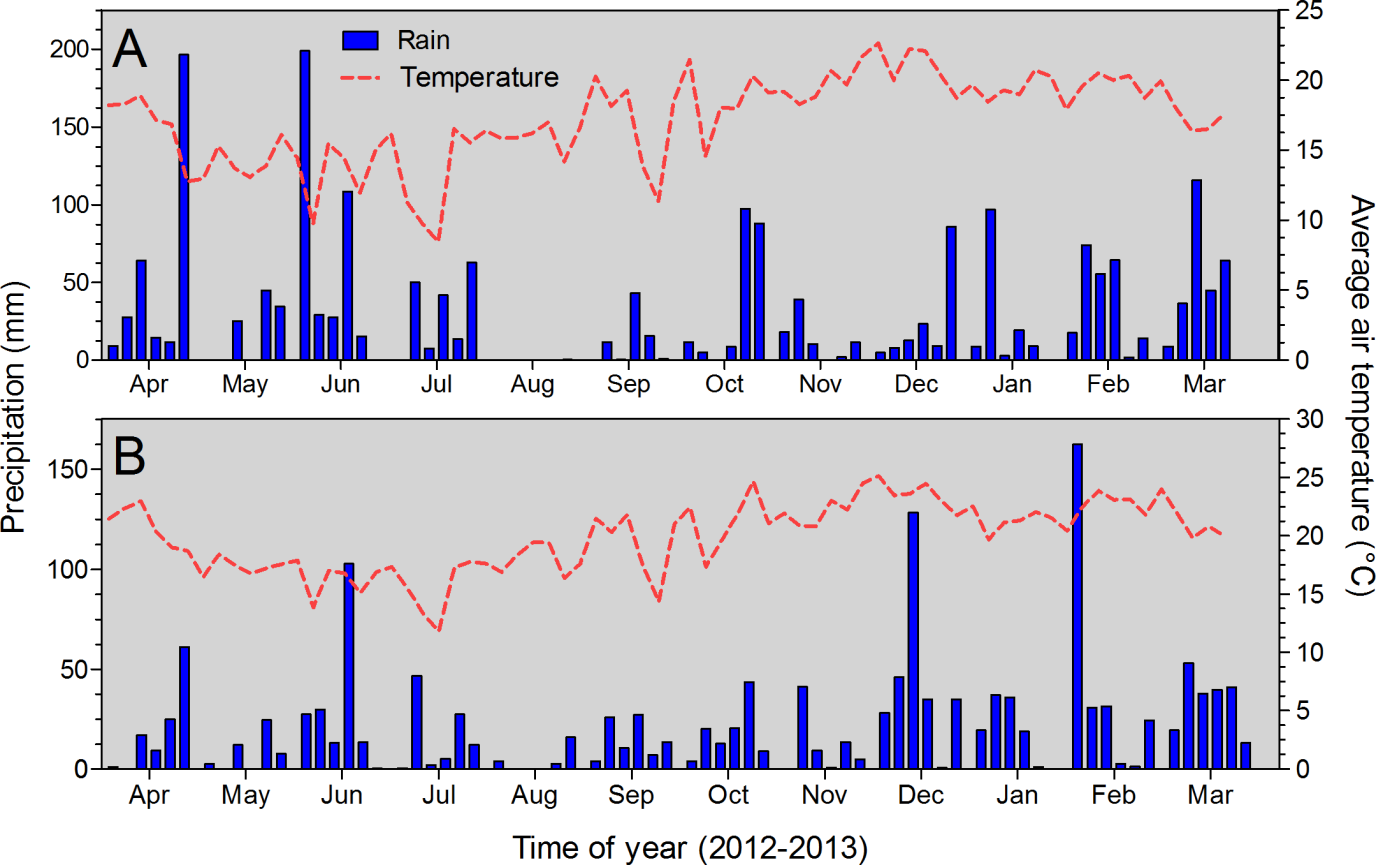
Seasonal precipitation and average air temperature during study period, from April 2012 to March 2013. (A) for site I, Guarapuava and (B) for site II, Taquarituba both located in Brazil. Blue bars referred to the precipitation, and red dashed lines represent the average seasonal temperature.

The studies were carried out on private land and the owners of the land gave permission to conduct the studies on these sites. Field experiments were conducted at two sites in 2012 season in the states of Paraná and Sao Paulo, Brazil. Each plot was comprised of seven rows, with a row spacing of 0.70 m for site I (Guarapuava, Paraná) and 0.45 m for site II (Taquarituba, São Paulo), by 10 m long. Before the onset of the study, soil test was conducted at the 0–20 cm soil depth. For each location, information related to soil type, oat planting date, corn planting, hybrid, and N fertilization timing was recorded (Table 1). Properties were as follows: pH of 5.1 and 5.5 units, a soil organic matter (SOM) of 51 and 40 g dm^-3^, P (resin as extractor) of 29 and 19 mg dm^-3^; K of 4.0 and 4.2 mmol_c_ dm^-3^, respectively, for sites I and II. Seasonal precipitation and temperature at each site are portrayed in Fig 1.

**Table 1:** Description of sites and management practices adopted.

Characteristic	Site I	Site II
County	Guarapuava	Taquarituba
Geographic coordinates	51°.66' E, 25° 52' S	49° 14' W, 23° 35' S
Altitude (m)	1100	630
Soil Type	Typic Oxisol	Typic Hapludalf
Soil texture in 0–20 cm layer		
Clay (g kg^-1^)	670	530
Silt (g kg^-1^)	230	270
Sand (g kg^-1^)	100	200
Precipitation (mm year^-1^)	2154	1683
Oat Planting Date	April, 25	May, 3
Oat	*Avena sativa*	*Avena strigosa*
Corn Planting Date	October, 1	November, 13
Corn Hybrids	AS1555	DKB390Hx
Plant population (plants ha^-1^)	70000	70000
Fertilizer N rate (kg ha^-1^)	180	150
N date application		
Oat	May, 18	May, 20
Pre-plant	Aug, 28	Oct, 30
Planting	Sep, 22	Nov, 12
V3 (three-leaf) corn	Oct, 16	Dec, 4
V3 / V6 (six-leaf) corn	Oct, 16 / Nov, 1	Dec, 4/16

### Crop management

Since 1982, the site I has been cultivated using no-tillage practices with a rotation soybean (*Glycine Max* L. Merr), corn, wheat (*Triticum aestivum* L.) and barley (*Hordeum vulgare* L.). In site II, last 25 years system included 15 years with pasture *Urochloa brizantha* and 10 years with a rotation soybean, corn, sorghum [*Sorghum bicolor* (L.) Moench] and oat under no-tillage. At both sites, oat (*Avena sativa* for Site I, and *Avena strigosa* for Site II) was planted over soybean residues and before corn planting time (Table 1). Oat crop did not receive any mineral fertilizer. Approximately 30 days before corn planting, oats were desiccated with glyphosate [N-(phosphonomethyl)glycine]. Except for N, phosphorous (P) and potassium (K) were supplied via fertilization at corn planting time, with 80 kg P_2_O_5_ ha^-1^ and 90 kg P_2_O_5_ ha^-1^ as triple superphosphate, and 113 kg K_2_O ha^-1^ and 90 kg K_2_O ha^-1^ as KCl for sites I and II, respectively.

### Experimental design and treatments

**Fig 2:**
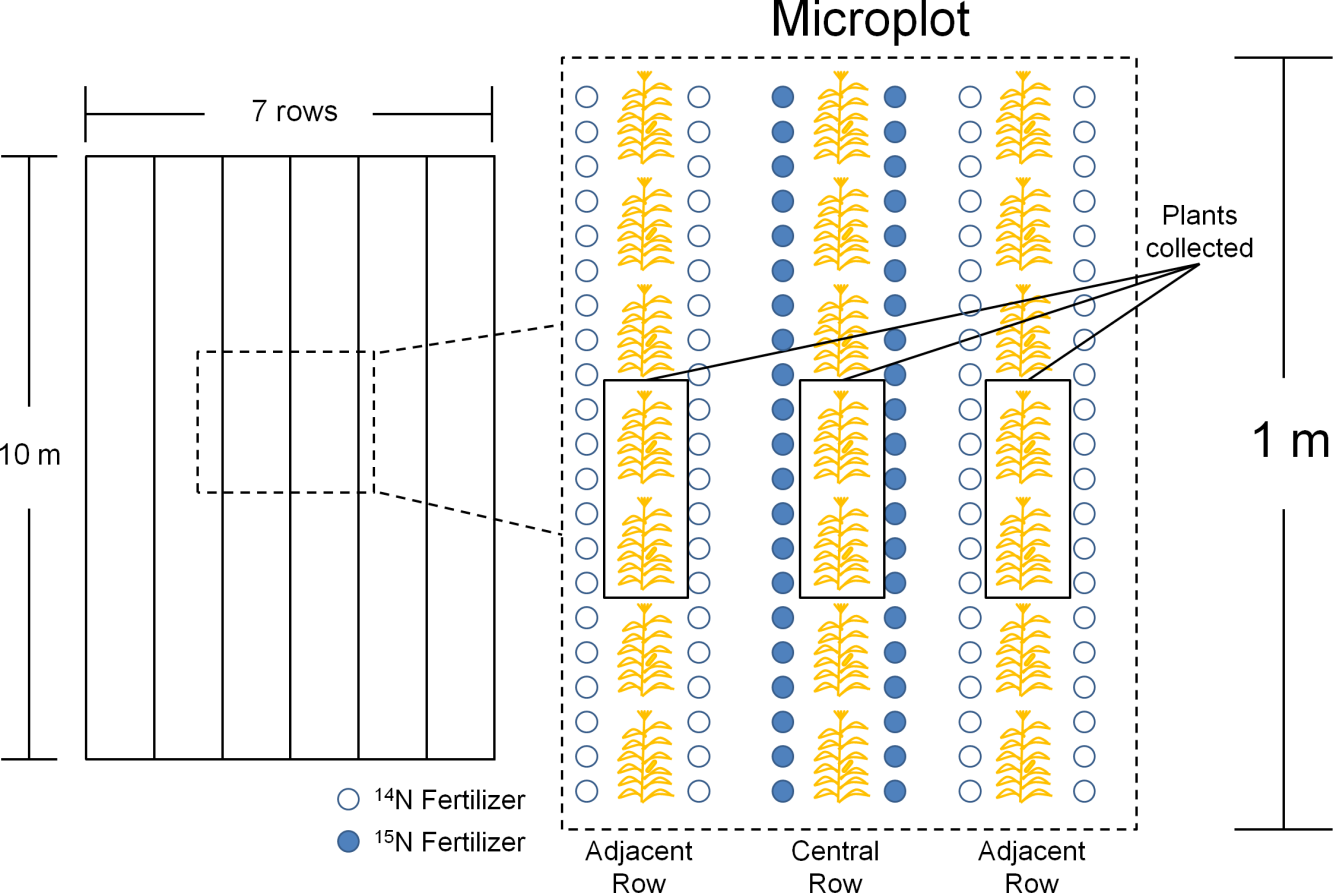
The isotopic labeled-N (^15^N) fertilizer was applied only to the central microplot row installed in each plot. Plants were sampled from the center part of the adjacent and the central rows.

Field studies were conducted in a randomized complete block (RCB) design with four replications. Treatments involved five N application timings: 1) at oat tillering; 2) at pre-plant over the oat residues (15 days before corn planting); 3) at corn planting; 4) in-season, at V3 growth stage (three-leaf; [29]); and 5) two split V3 and V6 (six-leaf) growth stages for corn. All fertilizer N application dates for sites I and II are presented in Table 1. Fertilizer N was applied to both left and right side of the corn rows (in-furrow) when the crop was planted/standing, or when applied to the oats the N fertilizer was placed in the same position where the corn rows were to be planted (oat tillering stage and oat residues) (Fig 2). The furrows were opened alongside the rows, approximately 0.08 m deep. A total of 180 kg N ha^-1^ for site I and 150 kg N ha^-1^ for site II were applied as urea. The target N rates selected to each site were applied according to Cantarella [30]. Final yield at all treatments was obtained by collecting 40 ears distributed throughout approximately 8 m and 12 m at three central rows for site I and II, respectively. For all individual ears harvested, yield and moisture were determined, with grain yield adjusted to 130 g kg^-1^ grain moisture content.

### Isotopic labeled-N determination (^15^N)

The isotopic ^15^N labeling was used to determine the amount of N fertilizer resulting in the corn plant, soil and oat residues. The fertilizer ^15^N application was identical to that described previously for the common fertilizer application in furrows (further details are presented in Fig 2). Urea enriched with ^15^N isotope (2.53% ^15^N atoms excess) was applied to the center of each plot according to the treatments, in microplots of 0.70 m^2^ in Guarapuava and 0.45 m^2^ in Taquarituba. To assess ^15^N recovery, two plants from the designated center row and four plants from the designated adjacent rows were collected in the center of the microplots within each plot (further details in schematic, Fig 2).

For ^15^N determination, corn plants were cut at the ground level and divided into shoot (ear, cob, stalk, leaves and tassel) and grain at physiological maturity. From the sampled plants, roots were dig up and collected utilizing rectangular trenches were up to 0.4 m deep under center row. Trenches dimensions were 1 m x 0.70 m for site I and 1 m x 0.45 m for site II according to the row spacing utilized in each site. Corn roots were carefully sieved (5 mm) and washed. Oat residues on the soil surface were also collected at corn harvest. Plant and residues samples collected from the microplots were dried for 72 h in a forced air circulation laboratory oven at 60°C to determine dry mass and then ground in a Wiley mill with a 2-mm mesh sieve.

In parallel to the biomass collection, soil samples from 0–20 and 20–40 cm were collected from the trenches dug up for the root sampling procedure. In the field, soil was homogenized by depth and packed in plastic bags. Soil samples from 40–60 cm soil depth were also collected using a probe positioned where center row was localized.

Abundance of ^15^N atoms and total N concentration were determined in an automated mass spectrometer ANCA-GSL N analyzer (Sercon Co. UK). The term N recovery efficiency (NRE) was used to express the percentage of the total N fertilizer recovered by corn plants. The N derived from fertilizer (NDFF) indicates the amount of N fertilizer in the corn or in soil, expressed in kg N ha^-1^. Fertilizer N recovery calculations and NRE were as follows:

$${NDFF}_{Central\;row}({kg\;ha}^{-1}\;of\;N)=\left[\frac{\alpha -\beta }{\gamma -\beta }\right]∙\;total\;N$$

$${NDFF}_{Adjacent\;row}\;({kg\;ha}^{-1}\;of\;N)=2∙\left[\frac{\alpha -\beta }{\gamma -\beta }\right]∙\;total\;N$$

$$Total\;NDFF\;({kg\;ha}^{-1}\;of\;N)={NDDF}_{Central\;row}+{NDFF}_{Adjacent\;row}$$

$${NDFF}_{Soil}({kg\;ha}^{-1}\;of\;N)=\left[\frac{\alpha -\beta }{\gamma -\beta }\right]∙\;total\;N$$

where NDFF is the amount of N derived from the fertilizer (kg ha^-1^), *α* is the abundance of ^15^N atoms in the sample (%), *β* is the natural abundance of ^15^N atoms (0.366%), *γ* is the abundance of ^15^N atoms in the fertilizer (2.53% atoms), and total N is the total of N (^15^N+^14^N) contained in the sample (kg ha^-1^).

$$NRE\;(\%)=\left(\frac{Total\;NDFF}{Fertilizer\;N\;Rate}\right)∙\;100$$

where NRE is the percentage of ^15^N recovered from the whole corn plant, total NDFF is the amount of N derived from the fertilizer (kg ha^-1^) and the fertilizer N rate is the rate of enriched fertilizer applied (kg ha^-1^).

The fertilizer fraction term portrays the N recover from the fertilizer (NDFF) regarding the total N content, and it was calculated using the equation:

$$Fertilizer\;Fraction=\left(\frac{\;NDFF}{\;plant\;N\;content-NDFF}\right)$$

where fertilizer fraction is an index of fertilizer N on plant N content.

### Statistical analysis

The response variables were submitted to normality test and homogeneity tests before the analysis of variance (ANOVA) was performed using PROC MIXED [31]. Site and block were considered as random factors, and treatments as fixed variables. If the null hypothesis was rejected, with significant treatment effect, Tukey mean comparison tests was performed at *P* < 0.05.

**Fig 3:**
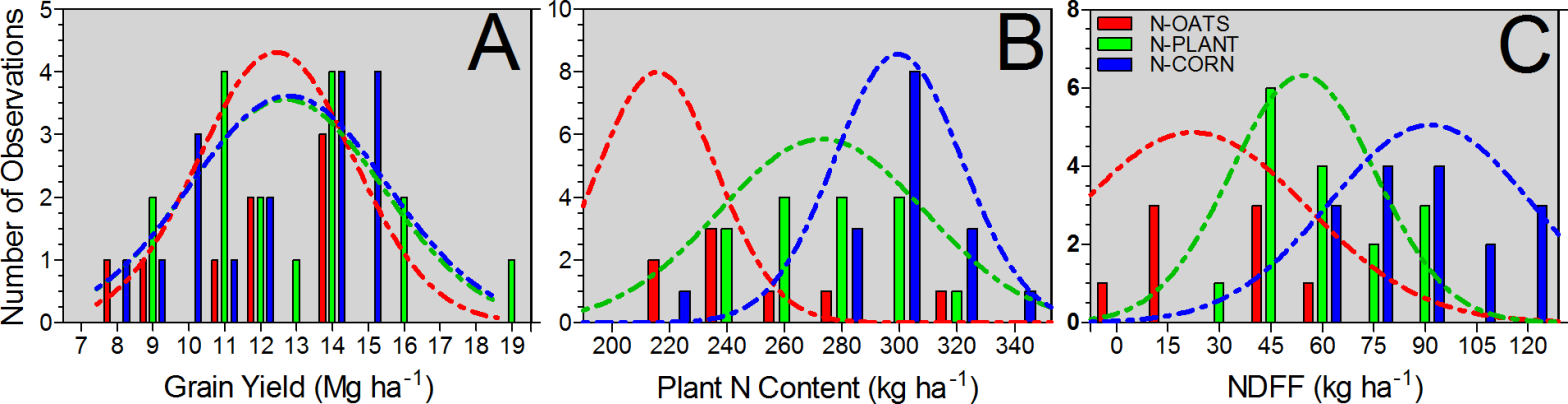
Data distribution for grain yield (A), plant N content (B) and NDFF (C) for the fertilizer N timing groups: fertilizer N applied to oats (N-OATS, red color), N applied at pre-plant and at planting time for corn (N-PLANT, green color), and in-season N added at V3 and at V3/V6 growth stages for corn (N-CORN, blue color).

Effect of fertilizer N application in corn was evaluated for yield, plant N content, NDFF, and NRE (Table 2). Since fertilizer N application influenced all N-related variables (plant N content, NDFF, and NRE) in a similar manner; a grouping was developed based on the results obtained from the Tukey mean comparison tests. Following this rationale, three groups were identified classifying the treatments in: i) fertilizer N applied to oats, herein termed as N-OATS; ii) N applied at pre-plant and at planting time for corn, herein termed as N-PLANT; and iii) in-season N added at V3 and at both V3/V6 growth stages (split-application) for corn, herein termed as N-CORN. For all three groups, descriptive statistics was implemented using “hist” function in R software [32]. Histograms were calculated for grain yield, plant N content, and NDFF (Fig 3A, 3B and 3C), with Gaussian models fitted for each N timing group {GraphPad Prism 6; [33]}. For the plant biomass and NDFF, and the NDFF and plant N content relationships linear components were tested [34] for each N timing group, and for each relationship best fitted model for each independent N timing group was compared with a global fit {GraphPad Prism 6; [31]} (Fig 3B and 3C).

**Table 2:** Grain yield, plant N content, N derived from fertilizer (NDFF) and ^15^N recovery (NRE, %) by corn for all N fertilization treatments.

Treatments	Grain yield	Plant N content	NDFF	NRE
	Mg ha^-1^	kg ha^-1^		(%)
Oat tillering	11.8	249 c	32 c	18 c
Pre-planting	12.8	276 bc	62 b	37 b
Corn planting	12.6	273 bc	59 b	35 b
Top dressing V3	12.7	287 ab	85 a	51 a
Top dressing V3/V6	12.1	302 a	92 a	55 a
Average	12.4	278	66	39
N application timings (NAT)	ns	**	***	***

ns = not significant

** significant at less than 0.1% and

*** significant at less 0.001% probability of error by the F test. Lowercase letters compare the means in the same column.

## Results

Corn grain yield was not affected by fertilization N timing (Table 2), presenting an average of 12.4 Mg ha^-1^. Contrastingly, plant N content, NDFF and NRE varied considerably among N timing. In summary, superior values were obtained when N was applied in-season rather than fallow or pre-plant N. In overall, plant N content ranged from 249 to 302 kg ha^-1^. The NDFF ranged from 32 to 92 kg ha^-1^, representing 18% and 55% of the N fertilizer applied (Table 2).

### Grain yield, plant N content, and NDFF

Distribution of grain yield, plant N content and NDFF are shown in Fig 3. Lack of statistical differences were documented for the mode and distribution for the yield parameter among all timing of N application (Fig 3A). For plant N content, data distribution portrayed three different groups related to timing of N application (Fig 3B). In-season N fertilization (N-CORN) presented an overall greater mode for plant N content followed by the application around planting (N-PLANT) and then to the oats (N-OATS). As observed with plant N content, the NDDF data distribution presented different modes as related to the timing of N application, ranking from high to low: N-CORN < N-PLANT < N-OATS (Fig 3C).

### Relationship between grain yield and plant N content

**Fig 4:**
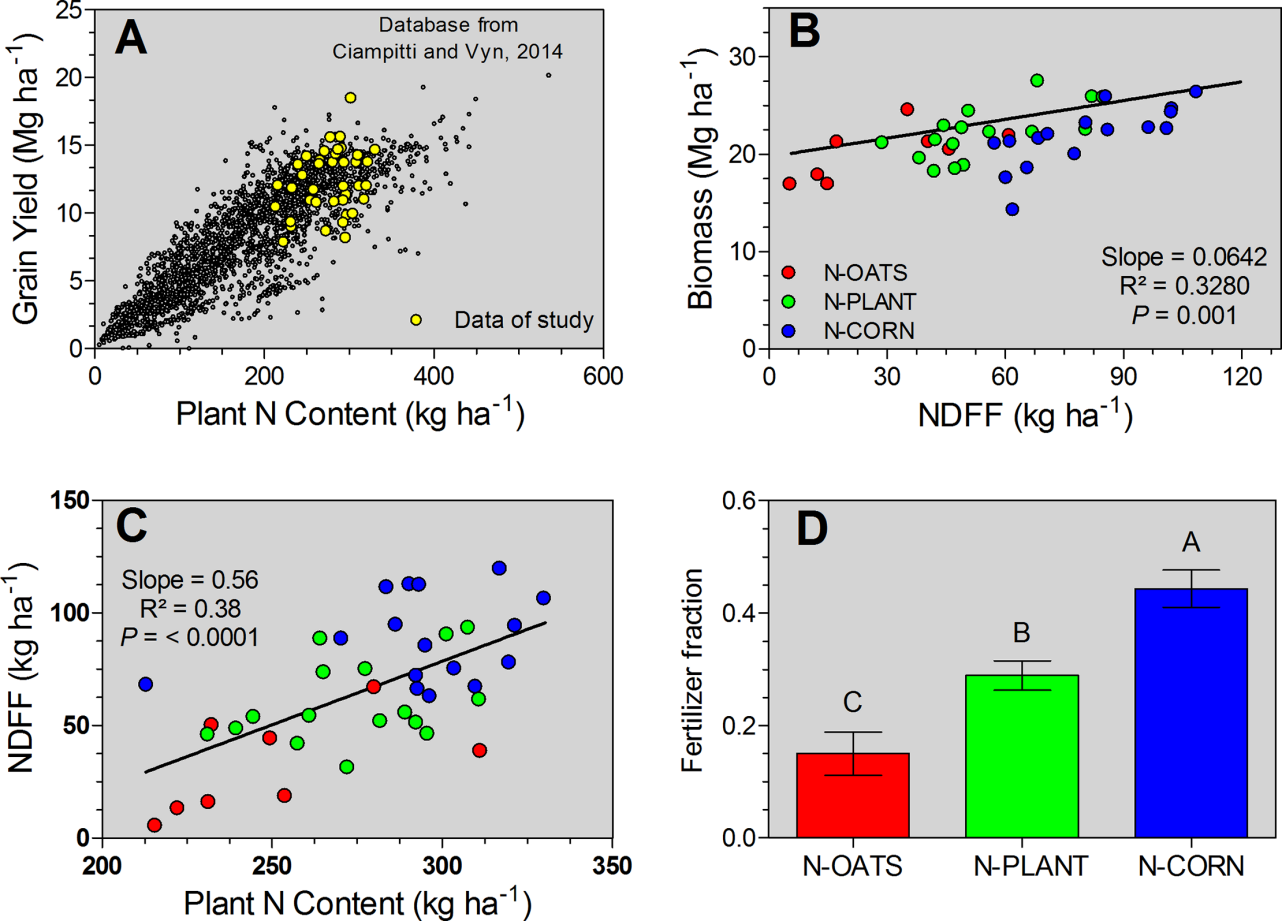
Relationship between grain yield and plant N content (A), biomass and NDFF (B), and NDFF, plant N content (C), and the overall fertilizer fraction on plant N content for the fertilizer N timing groups: fertilizer N applied to oats (N-OATS, red color; n = 8), N applied at pre-plant and at planting time for corn (N-PLANT, green color; n = 16), and in-season N added at V3 and V3/V6 growth stages for corn (N-CORN, blue color; n = 16).

The data points of grain yield to plant N content relationship from this current study were benchmarked against the historical corn and N synthesis dataset from the review published by Ciampitti and Vyn (2014). For the data collected in this study, yield increased at a lower rate relative to plant N content (Fig 4A). In overall, average plant N content per unit of yield was 22 kg N Mg^-1^. Despite the lack of effects in grain yield, plant biomass was linearly correlated to the NDFF, with both biomass and NDFF increasing as the fertilizer N application better matched (improved N synchrony) with the plant N demand (N-CORN treatment) (Fig 4B). Similarly, NDFF linearly increased as the plant N content rose at corn maturity, also related to the N timing with later applications increasing plant N content and NDFF (Fig 4C). In summary, the fertilizer fraction recovered in the plant N content increased as the N timing was delayed, more than two-fold when N-OATS was compared relative to the N-CORN (Fig 4D).

### NDFF distribution in soil, plant and final N budget

Soil distribution of NDFF was obtained immediately after corn harvest. Fertilizer N timing did not affect the final NDFF content with the exception of the shallowest soil layer, 0–20 cm (Table 3). The earliest N application (N-OATS) resulted in the largest NDFF content along all the soil layers, in overall 10 kg N ha^-1^ greater relative to the rest of the groups. Total NDFF content for the soil profile (0–60 cm) was approximately 10 kg ha^-1^ greater for the N-OATS relative to both N-PLANT and N-CORN groups (Table 3).

**Table 3:** Distribution of N derived from fertilizer (NDFF) at corn harvest at varying soil depths from 0–20 cm, 20–40 cm, 40–60 cm, and overall 0–60 cm.

Treatment	Soil Depth			
	0–20 cm	20–40 cm	40–60 cm	0–60 cm
	kg ha^-1^			
N-OATS	34.5	9.2 a	5.8 a	49.5 a
N-PLANT	26.9	6.2 b	6.1 a	39.2 b
N-CORN	27.2	7.7 ab	4.4 b	39.2 b
N application timings (NAT)	ns	*	*	*
CV (%)	29.6	37.7	26.7	26.6

^ns^ not significant

* significant at 5% probability of error by the F test.

**Fig 5:**
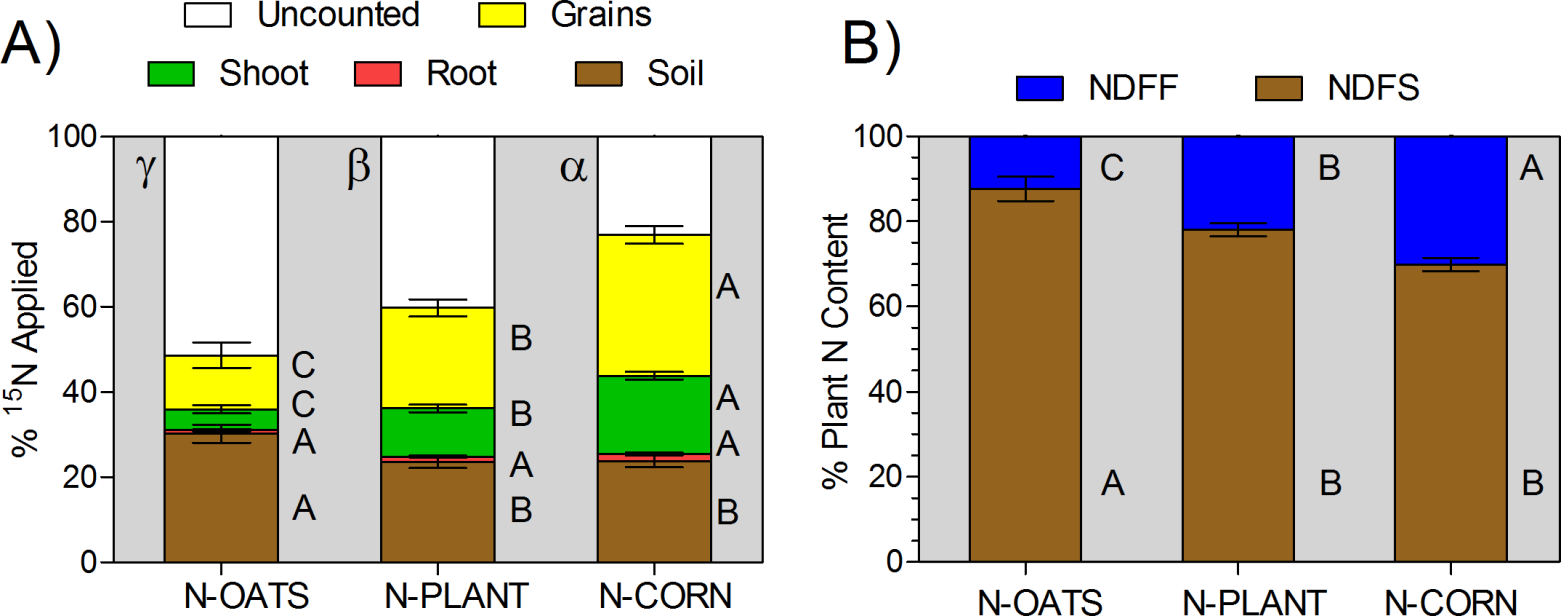
**Fate of N from fertilizer (A) and N source used by corn plants (B). Uppercase letters compare means of the components of the fate of N from fertilizer (A) or N source (B) within each fertilizer N timing level.** Greek letters compare total N fertilizer recovered in the columns. Bars represent the standard error for each treatment.

In the corn plant, fertilizer N recovery decreased as the N application was anticipated (Fig 5A). Final fertilizer N recovery **(soil-plant)** was 78%, 62% and 54% of the fertilizer N applied for the N-CORN, N-PLANT, and N-OATS groups, respectively. The fertilizer N recovery did not differ in the root organ, representing 1.3% of the total N applied. When N timing approached high N plant demand, fertilizer N recovery increased in both shoot and grain plant fractions. For the shoot organ, fertilizer N recovery was 18%, 11%, and 5% for N applied for the N-CORN, N-PLANT, and N-OATS treatments, respectively. For the grain fraction, fertilizer N recovery was 33%, 23% and 12% of the fertilizer N applied for the N-CORN, N-PLANT, and N-OATS groups, respectively (Fig 5A).

As related to the N sources utilized by corn plants, herein classified as NDFF from fertilizer and NDFS from native soil N, NDFS was resulted in the largest contributor to the final plant N content (Fig 5B). The NDFS contribution to plant N content was approximately 90% for the N-OATS, 78% for the N-PLANT, and only 70% for the N-CORN.

## Discussion

Lack of yield effect has been previously documented when fertilizer N timing was evaluated in years with adequate rainfall [17, 35, 36]. Increased plant N content and NDFF in N-CORN (when N was applied later in the season) was consistent with previous findings reported for fertilizing N timing studies [6, 37], as well as for ^15^N isotopic tracer fertilizer recovery values [12, 38, 39].

Improvement of plant N content and final NDFF in corn does not always result on higher yield [40–42]. For the current study, the yield-to-plant N content relationship (Fig 4A) portrayed a less than proportional change in yield as the plant N content increases. A previous review for corn yield-to-plant N content relationship documented a linear phase followed by a plateau, with a decrease in the yield-to-plant N content ratio as corn crop attained high-yielding environments, >10 Mg ha^-1^ [43].

Nevertheless, early N application could potentially affect grain yield under lower N rate as well as unfavorable environmental conditions for soil organic matter mineralization. A previous study [44] reported a 11% reduction in plant N content and a 42% reduction in biomass when low fertilizer N rates were applied under water deficit, portraying a tight relationship between N supply and yield.

For this current study, in-season fertilizer N applications did not impact yield but presented a positive and linear influence on biomass and plant N content (Fig 4B and 4C). Adequate crop N supply have a greater importance as the plant N demand increases, primarily from V10 (tenth-leaf) growth stage until mid-reproductive, R3 –milk stage [45]. Ning [46] documented that the plant N content accumulated during the post-silking period relative to the final N content achieved at maturity ranged from 11 to 43%. Therefore, superior plant N content and NDFF for the N-CORN treatment might be explained by the improved synchrony between supply-demand during the critical window for N uptake in corn, V10-R3 period. The latter was also reflected in a superior fertilizer recovery for the late N timing (Fig 5A).

In addition, N fertilize inputs affect the decomposer community, improving N mineralization from the SOM [22, 47]. Early N applications (before planting or in the cover crop) decreased utilization efficiency since N remains longer in the system and its more likely to be immobilized by soil microorganisms [26, 48, 49] or be subjected to losses [24, 25]. The latter could explain the lower plant N content achieved by N-OATS relative to the N-CORN group.

As for the recovery of the NDFF, superior recovery in the topsoil layer might be attributed to the greater microbial activity (more N immobilization) in the surface layer under no-tillage [50, 51]. Superior soil NDFF below 40 cm for N-OATS and N-CORN indicated leaching and increased in N movement when N fertilizer was applied at an early timing. These findings are in agreement with previous studies [6, 8, 36].

For plant fractions, NDFF distribution is in agreement (grain > shoot > root) with previously published studies on this topic [39, 42]. Nonetheless, NDFF proportion between grain and shoot fractions were affected among all the fertilizer N timing evaluated. As the N application timing was more in synchrony with the plant N demand, not only more NDFF was accounted for but also more N was documented in the grain relative to the shoot plant fraction (Fig 5A). Superior N in the grain can be the outcome of larger N remobilization and/or reproductive plant N uptake. In a review paper, Ciampitti et al. [45] reported high degree of correlation between vegetative N and N remobilization to the grain. Therefore, improving plant N nutrition before flowering by better synchrony between soil N supply and plant N demand can substantially improve reproductive N remobilization efficiency, allocating more N to the grain organ.

In overall at the system-level, better NRE was obtained as fertilizer N was applied at later timing also with a lower N non-recovery fraction. The latter is in agreement with studies performed under temperate conditions for corn or wheat wherein NRE decreased up to 15% when N added early in the season [16–18].

The influence of the fertilizer N timing can also be understood from the higher NDFS contribution in the early N application groups. The NDFS commonly overcame the NDFF contribution in cultivated crops [44, 52]. However, increased contribution of NDFS has often accompanied by more N asynchrony [13, 42, 53], as well as in the N-OATS and N-PLANT groups. In the current study, lack of yield differences among fertilizer N timing groups is primarily explained by superior NDFS contribution as the NRE diminished. The latter is tightly connected to the soil N mineralization potential. Nonetheless, it should be warranted that early N application under subtropical conditions is not a viable option since fertilizer NRE is reduced, increasing the non-N recovered fraction.

## Conclusions

Timing of fertilizer N application substantially impacted NRE and plant N content, with NRE and plant N content improving as the application of N was better synchronized with the plant N demand. Yield was not statistically influenced by fertilization N timing most likely due to the larger NDFS contribution as NRE decreased for the early N timing treatments. Lastly, approximately more than 10% of the fertilizer applied was retained within the soil profile (0–60 cm), and more specifically below 40 cm, indicated potential leaching.

This study suggests larger potential for fertilizer losses and downward fertilizer N movement in the soil profile due to early N applications. Despite the larger NDFS supply and the lack of yield effect; the best compromise to obtain superior NRE and minimize potential N losses, diminishing the fertilizer N environmental footprint, is to improve synchrony between soil N supply and plant N demand.

## Supporting information

S1 File Grain yield, plant N content and N derived from fertilizer (NDFF) among all the treatments. (XLSX)

## Abbreviations

N: Nitrogen
NRE: N recovery efficiency
NDFF: N derived from fertilizer
SOM: Soil organic matter
